# *Panax notoginseng* transcription factor WRKY15 modulates resistance to *Fusarium solani* by up-regulating *osmotin-like protein* expression and inducing JA/SA signaling pathways

**DOI:** 10.1186/s12870-023-04373-x

**Published:** 2023-07-17

**Authors:** Linlin Su, Lilei Zheng, Hanlin Wang, Yuan Qu, Feng Ge, Diqiu Liu

**Affiliations:** 1 Faculty of Life Science and Technology, Kunming University of Science and Technology, Kunming, 650500 Yunnan China; 2 Yunnan Provincial Key Laboratory of Panax notoginseng, Kunming, 650500 Yunnan China

**Keywords:** *Panax notoginseng*, WRKY transcription factor, Osmotin, Jasmonic acid, Salicylic acid, *Fusarium solani*

## Abstract

**Background:**

*Panax notoginseng* (Burk) F. H. Chen is a valuable traditional Chinese medicinal plant, but its commercial production is seriously affected by root rot caused by some pathogenic fungi, including *Fusarium solani*. Nevertheless, the genetic breeding for disease resistance of *P. notoginseng* remains limited. The WRKY transcription factors have been revealed to play important roles in plant defense responses, which might provide an inspiration for resistance improvement in *P. notoginseng*.

**Results:**

In this study, the regulatory mechanism of transcription factor PnWRKY15 on *P. notoginseng* resistance to *F. solani* infection was revealed. The suppressed expression of *PnWRKY15* via RNA interference increased the sensitivity of *P. notoginseng* to *F. solani* and decreased the expression levels of some defense-related genes, including *PnOLP1*, which encodes an osmotin-like protein that confers resistance to *F. solani*. Ectopic expression of *PnWRKY15* in the model plant tobacco significantly enhanced the resistance to *F. solani*. Moreover, the transcriptome sequencing analysis discovered that some pathogenesis-related genes were expressed at higher levels in the *PnWRKY15*-overexpressing tobacco than that in the wild-type tobacco. In addition, the jasmonic acid (JA) and salicylic acid (SA) signaling pathways were evidently induced by *PnWRKY15*-overexpression, that was evidenced by that the JA and SA contents were significantly higher in the *PnWRKY15*-overexpressing tobacco than that in the wild-type. Furthermore, PnWRKY15, which was localized in the nucleus, can trans-activate and up-regulate *PnOLP1* expression according to the EMSA, yeast one-hybrid and co-expression assays.

**Conclusions:**

PnWRKY15 contributes to *P. notoginseng* resistance to *F. solani* by up-regulating the expression of resistance-related gene *PnOLP1* and activating JA/SA signaling pathways. These findings will help to further elucidate the transcriptional regulatory mechanism associated with the *P. notoginseng* defense response to *F. solani*.

**Supplementary Information:**

The online version contains supplementary material available at 10.1186/s12870-023-04373-x.

## Background

WRKY transcription factors (TFs), which belong to one of the largest TF families, contain a highly conserved DNA-binding heptapeptide (WRKYGQK) and a zinc finger motif. They actively respond to pathogen infections and reprogram the transcriptome to trigger the plant immune response. The expression levels of most of the identified *WRKY* genes in pepper (*Capsicum annuum*) are up-regulated in plants inoculated with *Phytophthora capsicum* and pepper mottle virus [1]. A total of 42 *Akebia trifoliata WRKY* genes have been characterized, of which *AktWRKY11*/*18*/*21*/*31*/*47- 2*/*51*/*65*/*70*/*74* were up-regulated in response to a *Colletotrichum acutatum* infection [2]. Additionally, 23 *WRKY* genes have been identified in the *Botrytis*-resistant (*Botrytis elliptica* and *Botrytis cinerea*) species *Lilium regale*; the ectopic expression of *LrWRKY4* and *LrWRKY12* in *Arabidopsis thaliana* leads to enhanced resistance to *B. cinerea* [3].

WRKY TFs bind to the *cis*-element W-box [(C/T)TGAC(T/C)] in target gene promoters to regulate expression [4]. Changes to any of the nucleotides in the core sequence (TGAC) greatly hinder the ability of WRKY TFs to bind to the W-box *cis*-element [5]. Apple (*Malus domestica*) WRKY31 binds specifically to the W-box2 *cis*-element in the promoter of *MdHIR4*, which encodes a negative regulator in pathogenic resistance to *Botryosphaeria dothidea*, and then represses *MdHIR4* expression, thereby enhancing apple resistance to *B. dothidea* [6]. Four cotton (*Gossypium hirsutum*) group IIc WRKY TFs bind directly to the W-box element in the *GhMKK2* promoter to positively regulate its expression, leading to increased resistance to *Fusarium oxysporum* f. sp. *vasinfectum* [7].

Earlier research indicated that WRKY TFs and signal transduction pathways associated with various hormones, including jasmonic acid (JA), ethylene (ET), salicylic acid (SA), and abscisic acid (ABA), cooperatively mediate plant stress responses [4]. The expression of *WRKYs* in lotus (*Nelumbo Nucifera*) roots was regulated by JA and SA signaling, especially for *NnWRKY40a* and *NnWRKY40b*, both of which were significantly induced after JA treatment [8]. In oil palm (*Elaeis guineensis*), *WRKY* gene expression is induced in response to various concentrations of ABA, methyl jasmonate (MeJA), SA, and H_2_O_2_ following an exposure to drought stress [9]. Notably, JA and SA are critical hormones mediating signal transduction after a pathogen infection [10]. The JA signaling pathway generally affects the defense response to herbivores and necrotic pathogens, whereas the SA signaling pathway usually mediates the defense response to biotrophic pathogens [11]. However, the cross-talk between different hormone (e.g., JA and SA) signaling pathways is relatively complex. The regulatory effects of JA and SA signaling pathways on plant immune responses to pathogens are antagonistic or synergistic [12].

*Panax notoginseng* (Burk) F. H. Chen is an important medicinal plant that is traditionally used to promote blood circulation, stop bleeding, and enhance the human immune system [13]. The medicinal materials derived from *P. notoginseng* are mainly produced in Wenshan prefecture, Yunnan province, China. As a perennial species, 3-year-old *P. notoginseng* plants may be used to produce medicine. Unfortunately, *P. notoginseng* plants are susceptible to many pathogens during their long growth period. For example, *Fusarium* species, including *Fusarium solani*, are responsible for *P. notoginseng* root rot, which can significantly decrease plant yield and quality [14]. The application of fungicidal chemicals is the traditional method used to control root rot, but the available chemicals have limited efficacy. Moreover, these chemicals may pollute the soil environment and result in accumulation of medicinal products unsafe for human consumption. To develop an environmentally friendly and effective method for controlling *P. notoginseng* root rot, the mechanism underlying the plant defense response to the disease must be elucidated.

Previous studies confirmed that an exogenous MeJA treatment increases the resistance of *P. notoginseng* to *F. solani*. Furthermore, a series of JA-responsive *F. solani* resistance-related genes in *P. notoginseng* has been isolated, including *osmotin-like protein* (*PnOLP1*) [15]. The *PnOLP1* expression level is significantly up-regulated by MeJA and SA treatments as well as by *F. solani* infections. The encoded protein, which is localized in the cell wall, can inhibit *F. solani*, *F. oxysporum*, and *Fusarium graminearum* mycelial growth in vitro. The ectopic expression of *PnOLP1* in tobacco (*Nicotiana tabacum*) reportedly increases the resistance to *F. solani* [15]. The resistance induced by the application of exogenous MeJA is accompanied by the activated expression of many *WRKY* genes, including *PnWRKY9* and *PnWRKY15* [16]. Additionally, PnWRKY9 and the JA signaling pathway synergistically enhance root rot resistance by regulating the expression of a defensin gene, *PnDEFL1* [17]. The WRKY TF-mediated network regulating plant defense responses to pathogens is extremely complex. Therefore, in this study, we explored the molecular basis of PnWRKY15 in response to an infection by *F. solani* in *P. notoginseng*.

## Results

### *P. notoginseng* WRKY15 is a nuclear protein

**Fig. 1:**
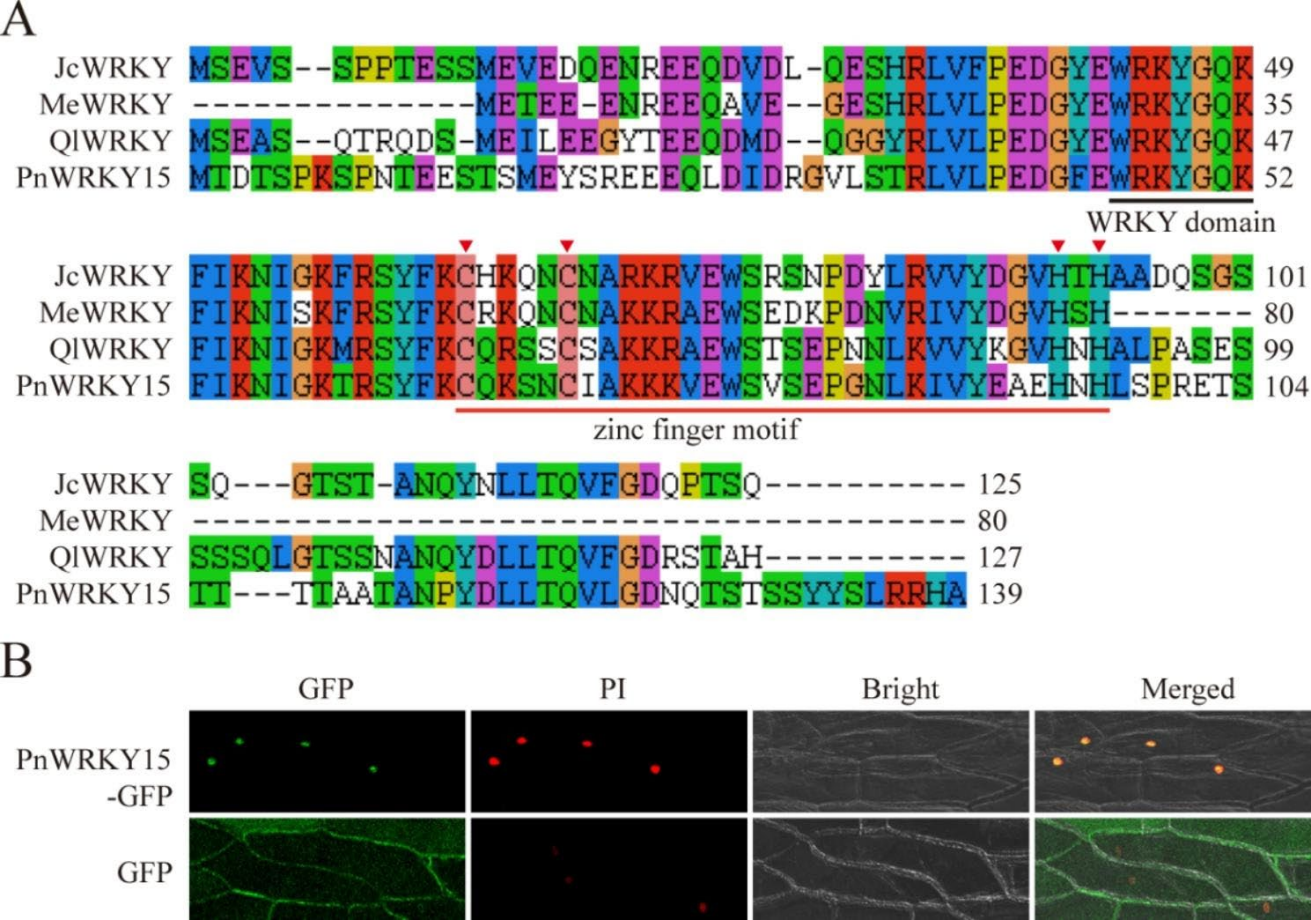
The sequence analysis and subcellular localization of PnWRKY15. **(A)** The multiple sequence alignment of PnWRKY15 and three homologous WRKYs. **(B)** The expression of *PnWRKY15-GFP* and *GFP* empty vector in onion epidermal cells. GFP: green fluorescent protein; PI: propidium iodide; Bright: white light field; Merged: superposition of fluorescent field and white light field

The *P. notoginseng* WRKY gene family was identified in [17]. In the current study, we focused on the regulatory effect of PnWRKY15 on the defense response to root rot. The *PnWRKY15* cDNA was 960 bp long, with a 420 bp open reading frame (ORF) encoding a protein comprising 139 amino acid residues. The deduced protein (PnWRKY15) was predicted to be 15.87 kDa, with an isoelectric point of 7.51. The sequence analysis revealed PnWRKY15 contains a conserved heptapeptide (WRKYGQK), which is followed by a zinc finger motif (C-X4-5-CX22-23-H-X1-H; X refers to any amino acid) (Fig. 1A). In addition, PnWRKY15 is highly homologous to *Jatropha curcas* WRKY45 (XP_012075800.1), *Quercus lobata* WRKY3 (XP_030954519.1), and *Manihot esculenta* WRKY28 (XP_021596011.1), with sequence identities of 87%, 75%, and 69%, respectively. The *PnWRKY15-GFP* fusion cascade was expressed in onion epidermal cells to clarify the subcellular localization of PnWRKY15. The fluorescence of PnWRKY15-GFP was detected in the nucleus. The subcellular localization result was confirmed by the colocalization of propidium iodide (PI, a nucleus-staining dye) (Fig. 1B).

### Down-regulation of *PnWRKY15* expression via RNA interference (RNAi) increased the susceptibility of *P. notoginseng* to *F. solani*

**Fig. 2:**
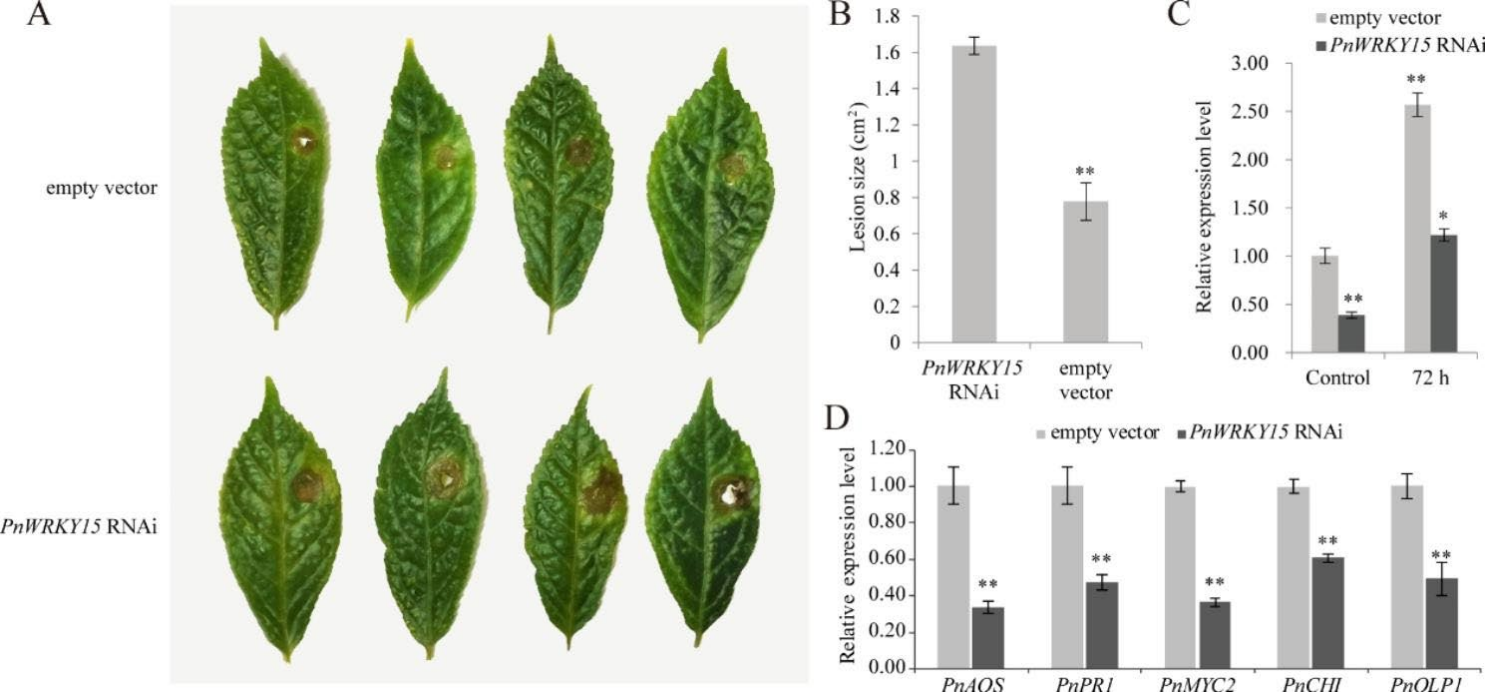
Analysis of transient expression of phellsgate2-*PnWRKY15* in *P. notoginseng* leaves. **(A)** The symptoms of *P. notoginseng* leaves after *Fusarium solani* inoculation, in which the *PnWRKY15* RNAi vector and the empty RNAi vector were expressed, respectively; **(B)** The analysis of diseased area in *P. notoginseng* leaves; **(C, D)** The expression levels of *PnWRKY15* and defense related genes in *PnWRKY15*-RNAi and control *P. notoginseng* leaves. The results were shown as average values calculated from three replicates and the significance was determined by the Student’ s *t*-test (*: *P* < 0.05; **: *P* < 0.01)

There is currently a lack of a practical and efficient method for the genetic transformation in *P. notoginseng*. An RNAi fragment targeting *PnWRKY15* was transiently expressed in the young leaves of *P. notoginseng* plants to determine whether the gene is involved in defense response to *F. solani*. The quantitative real-time polymerase chain reaction (qRT-PCR) data indicated that the *PnWRKY15* expression level was approximately 50% lower in the *P. notoginseng* leaves expressing the RNAi fragment than that in the control *P. notoginseng* leaves carrying the empty vector (Fig. 2A), reflecting a successful RNAi-based down-regulation of *PnWRKY15* expression in *P. notoginseng* leaves. After inoculation with *F. solani*, *PnWRKY15* expression was induced in the RNAi plants, but its expression level was still significantly lower than that in the control plants. In terms of the symptoms caused by the *F. solani* infection, the decayed area was larger in the leaves of RNAi plants than that of the control (Fig. 2B-C). The expression levels of JA/SA signaling-related genes, including *PnAOS*, *PnMYC2*, and *PnPR-1*, as well as two pathogenesis-related (PR) genes (*PnCHI* and *PnOLP1*), decreased in the RNAi *P. notoginseng* plants (Fig. 2D). Accordingly, the down-regulated expression of *PnWRKY15* increased the susceptibility of *P. notoginseng* to *F. solani* and suppressed the expression of some disease resistance related genes. These findings suggest that *PnWRKY15* encodes a positive regulator in *P. notoginseng* defense response against *F. solani*.

### Ectopic expression of *PnWRKY15* in tobacco enhanced the resistance to *F. solani*

**Fig. 3:**
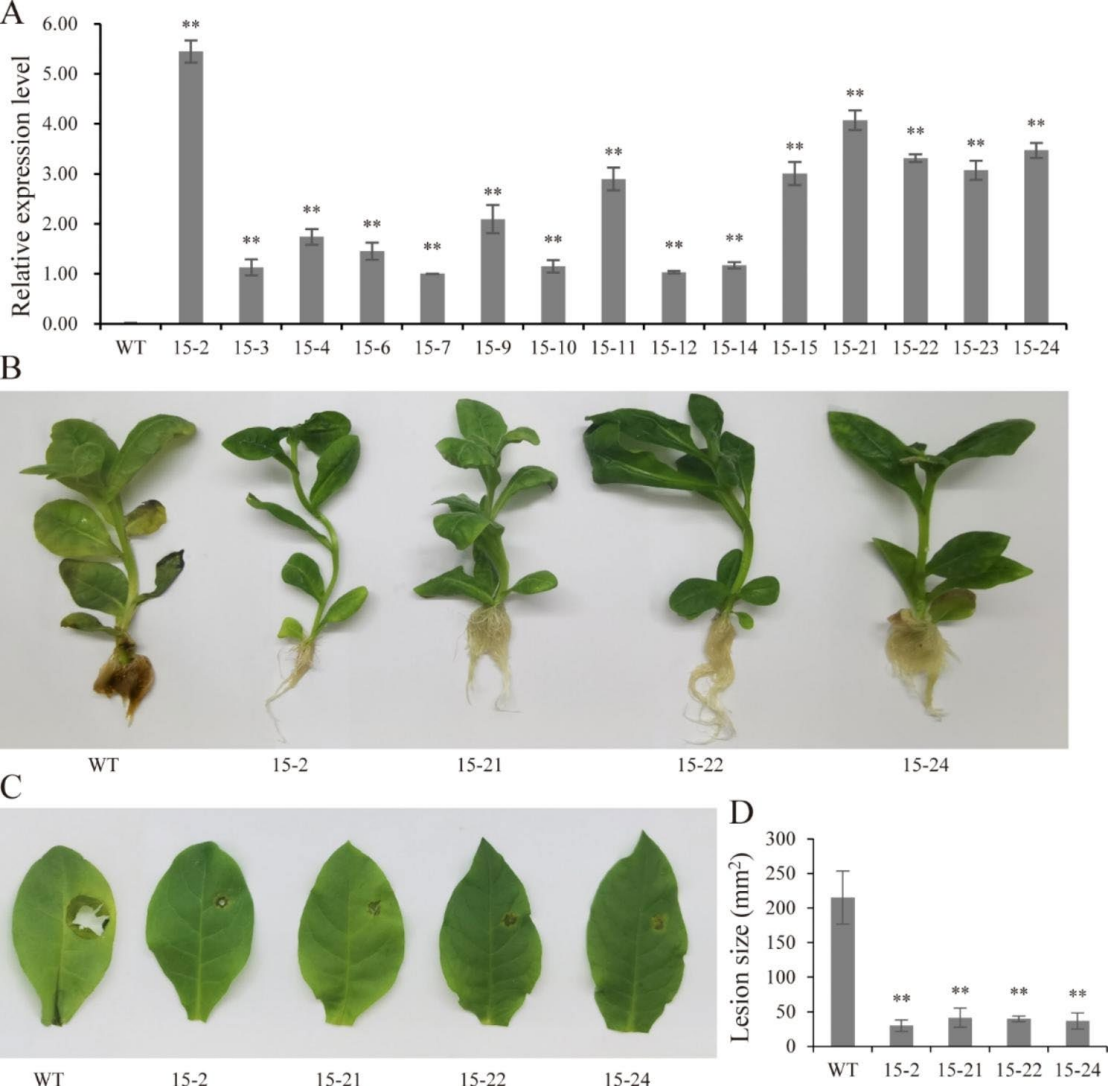
Resistance analysis of *PnWRKY15*-OE tobacco lines. **(A)** Transcription level of *PnWRKY15* in transgenic tobacco lines. WT: wild-type tobacco; 15 - 2/3/4/6/7/9/10/11/12/14/15/21/22/23/24: *PnWRKY15*-OE lines. **(B)** The root inoculation assay revealed the enhanced resistance of *PnWRKY15* transgenic tobacco lines to *Fusarium solani* infection. **(C)** The symptoms in tobacco leaves after inoculation with *F. solani* for one week. **(D)** The analysis of diseased area in *tobacco* leaves. The results were shown as average values calculated from three replicates and the significance was determined by the Student’ s *t*-test (*: *P* < 0.05; **: *P* < 0.01)

An exogenic gene can be stably expressed in tobacco through *Agrobacterium* mediated genetic transformation, which is a more reliable method to accurately uncover the function of that gene. In addition, the *F. solani* causes root rot in tobacco [18]. Therefore, the *PnWRKY15* gene was ectopically expressed in the model plant tobacco to verify its function. The T_2_ generation of *PnWRKY15-*overexpressing (OE) tobacco lines were developed and examined. Firstly, 15 tobacco lines were selected for the analysis of *PnWRKY15* expression. The qRT-PCR data confirmed that *PnWRKY15* was significantly over expressed in all of the tested OE lines, although the transcript levels varied (Fig. 3A). More specifically, the *PnWRKY15* expression level was highest in line 15 - 2, with a relative expression level of 5.44. Other OE lines, including 15 - 11/-15/-21/-22/-23/-24, had a relative expression level of 2.90, 3.01, 4.07, 3.31, 3.07, and 3.47, respectively.

The resistance of the *PnWRKY15-*OE tobacco lines to *F. solani* was subsequently evaluated. Four T_2_ *PnWRKY15*-OE tobacco lines (15 - 2/-21/-22/-24) were tested. After a 7-day incubation of tobacco roots with *F. solani*, the leaves of wild-type (WT) tobacco turned curl, yellow, and withered. Moreover, the roots were visibly black and rotten. In contrast, the *PnWRKY15*-OE tobacco lines kept healthy growth (Fig. 3B). In addition, after 7 days inoculation with *F. solani*, the WT tobacco leaves appeared with obvious yellowing and decay, whereas the leaves from four *PnWRKY15*-OE tobacco lines were only slightly yellow or normal (i.e., no decay) (Fig. 3C). The lesions caused by the *F. solani* infection were approximately 8-times larger on the WT leaves than that of *PnWRKY15*-OE leaves (Fig. 3D). Hence, ectopic expression of *PnWRKY15* in tobacco significantly increased the resistance to *F. solani*.

### The tobacco transcriptome profile was altered by the overexpression of *PnWRKY15*

**Fig. 4:**
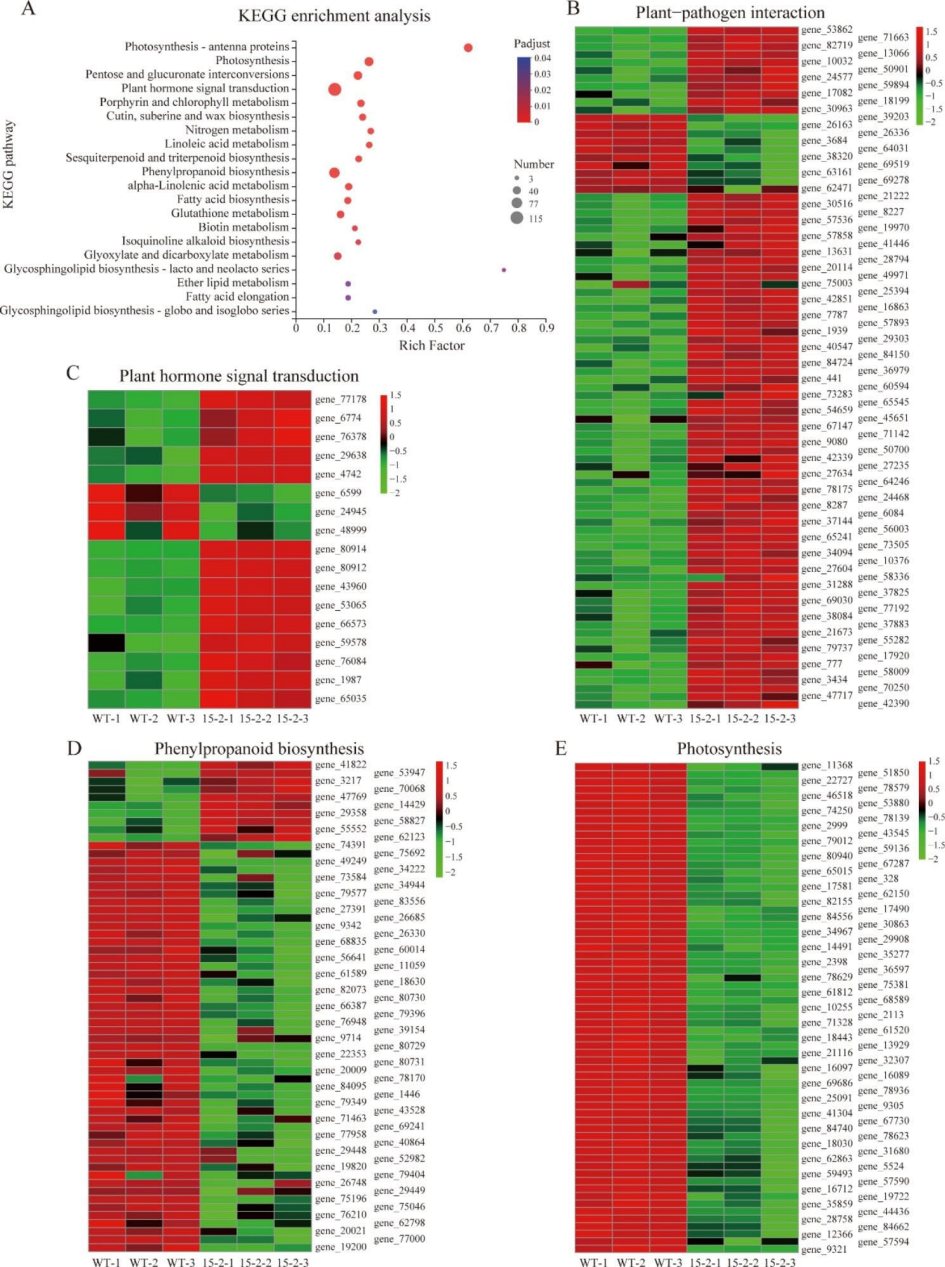
Enrichment analysis of KEGG pathway based on the RNA-seq data of *PnWRKY15* transgenic tobacco with the wild-type tobacco as a control. **(A)** KEGG bubble map; **(B-E)** The heat maps of differentially expressed genes in the pathways including plant pathogen interaction, plant hormone signal transduction, phenylpropanol biosynthesis, and photosynthesis. WT-1/-2/-3: wild-type tobacco; 15-2-1/-2/-3: *PnWRKY15* transgenic line 15 -2

To clarify the regulatory effect of PnWRKY15, one of the *PnWRKY15*-OE tobacco lines (15 - 2) was selected for a transcriptome sequencing (RNA-seq) analysis. Compared with the WT tobacco, 6,820 genes were differentially expressed in the *PnWRKY15*-OE tobacco. Of these differentially expressed genes (DEGs), 2,245 and 4,575 were up-regulated and down-regulated, respectively. The enriched Kyoto Encyclopedia of Genes and Genomes (KEGG) pathways among the DEGs are presented in Fig. 4A and Supplementary Material 1: Table S1. The most significantly enriched KEGG pathways were plant–pathogen interaction, plant hormone signal transduction, phenylpropanoid biosynthesis, and photosynthesis (Fig. 4B–E). The expression of several unigenes involved in plant-pathogen interactions was induced in the *PnWRKY15*-OE tobacco line (Fig. 4B). The expression levels of key genes related to JA biosynthesis, including genes encoding allene oxide synthase (gene_80914, gene_80912, gene_43960, and gene_53065), were much higher in the *PnWRKY15*-OE tobacco than that of WT tobacco (Fig. 4C, Supplementary Material 1: Table S2). In contrast, *Jasmonate ZIM-domain* genes (gene_6599, gene_24945, and gene_48999), which are associated with the repression of JA signal transduction, were expressed at lower levels in the *PnWRKY15*-OE tobacco than that of the WT tobacco. Moreover, the expression levels of the *TGA* genes (gene_76084, gene_1987, and gene_65035), which encode TFs that regulate SA signaling, were clearly up-regulated in the *PnWRKY15*-OE tobacco (Fig. 4C). Additionally, the expression of genes related to phenylpropanoid biosynthesis differed significantly between the WT and *PnWRKY15*-OE tobacco samples, including genes encoding phenylalanine deaminase (gene_62123), cinnamyl alcohol dehydrogenase (gene_3217), and peroxidase (gene_47769, gene_14429, gene_29358, gene_58827, and gene_55552) (Fig. 4D). Furthermore, multiple photosynthesis-related genes had down-regulated expression levels (Fig. 4E). These results indicated that ectopic expression of *PnWRKY15* had a wide regulation on tobacco gene expression.

### Overexpression of *PnWRKY15* in tobacco activated the JA/SA signaling pathways and plant–pathogen interactions

**Fig. 5:**
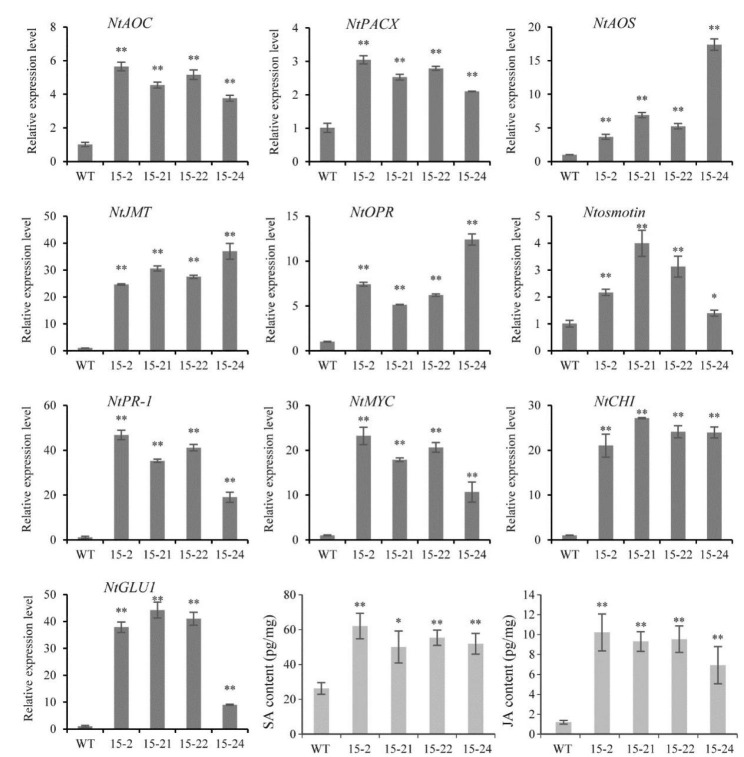
Transcription levels of defense related genes and determination of JA/SA content in *PnWRKY15* transgenic tobacco. WT: wild-type tobacco; 15 - 2/-21/-22/-24: *PnWRKY15* transgenic tobacco lines. The results were shown as average values calculated from three replicates and the significance was determined by the Student’s *t*-test. *: *P* < 0.05; **: *P* < 0.01

The RNA-seq analysis revealed the extensive changes in the transcriptome of the *PnWRKY15*-OE tobacco. Therefore, the relative expression levels of many genes associated with JA/SA signaling pathways and plant-pathogen interactions were analyzed by qRT-PCR. Four *PnWRKY15*-OE tobacco lines (15 - 2/-21/-22/-24) were included in this experiment (Fig. 5). Five JA biosynthesis-related genes (*NtAOC*, *NtPACX*, *NtAOS*, *NtJMT*, and *NtOPR*) were expressed at higher levels in the three *PnWRKY15*-OE tobacco lines than that of WT tobacco, as was *NtMYC*, which encodes a TF that positively regulates JA signaling. Some PR genes, such as *NtGLU1*, *NtPR1*, *Ntosmotin*, and *NtCHI*, were significantly more highly expressed in the *PnWRKY15*-OE tobacco lines than that of WT. Of these genes, *NtPR1* is a marker gene of the SA signaling pathway [19].

On the basis of above gene expression analysis, JA and SA contents in the *PnWRKY15*-OE tobacco were measured (Fig. 5). The average SA/JA contents of *PnWRKY15*-OE tobacco were 2.1-times and 7.5-times higher than that of the WT tobacco, respectively. The significant increases in the JA and SA contents in the *PnWRKY15*-OE tobacco indicated that PnWRKY15 may activate the JA and SA signaling pathways in tobacco, both of which were reportedly important pathways in plant disease resistance.

### PnWRKY15 trans-activated an *F. solani* resistance-related gene, *PnOLP1*

Down-regulation of *PnWRKY15* through RNAi significantly suppressed *PnOLP1* expression (Fig. 2B). Moreover, overexpression of *PnWRKY15* induced the expression of PR genes in tobacco, including *Ntosmotin* (Fig. 5). We previously identified *PnOLP1* as an *F. solani* resistance-related gene [15]. It was reasonable to verify whether *PnWRKY15* had a direct regulation on *PnOLP1* transcription. Therefore, the *PnOLP1* promoter (PPnOLP1) was cloned to determine whether PnWRKY15 can directly bind to PPnOLP1. The PPnOLP1 sequence (965 bp) is shown in Supplementary Material 2. It has a W-box (TTGACC) element (− 31 to − 37 bp) (Supplementary Material 1: Table S3), as well as IAA- and ABA-responsive elements, high-salt- and dark-responsive elements, and a MYB-binding site.

**Fig. 6:**
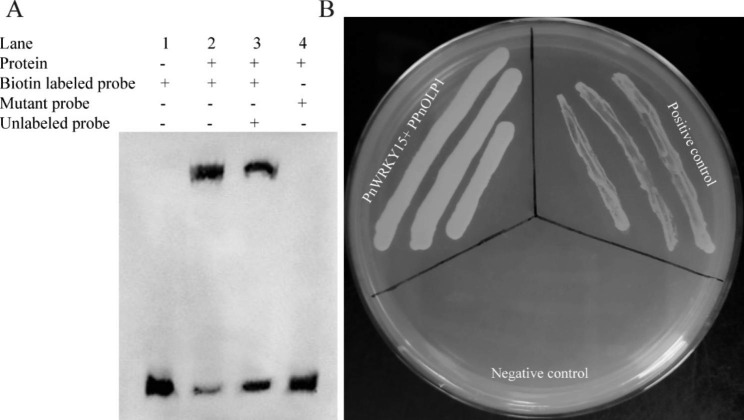
PnWRKY15 specifically bound to W-box from *PnOLP1* promoter and has transcriptional activation on *PnOLP1*. **(A)** The recombinant PnWRKY15 specifically bound to W-box from *PnOLP1* promoter. Lane 1: reaction solution containing only biotin labeled probes;Lane 2: reaction solution containing biotin labeled probes and recombinant PnWRKY15; Lane 3: reaction solution containing biotin labeled probes, unlabeled competitive probes and recombinant PnWRKY15; Lane 4: reaction solution containing biotin labeled mutant probes and recombinant PnWRKY15. Original image of Fig. 6A was shown in the Supplementary Material 4. **(B)** Analysis of the trans-activation of PnWRKY15 on *PnOLP1* promoter (PPnOLP1). The pGADT7-*PnWRKY15* prey vector and pAbAi-PPnOLP1 bait vector were co-transformed into yeast cells, which grew on SD/−Leu/AbA (200 ng/mL) medium, indicating that PnWRKY15 trans-activated PPnOLP1. PnWRKY15 + PPnOLP1: pGADT7-*PnWRKY15* and PPnOLP1-pAbAi co-transformed yeast cells; Positive control: pGADT7-Rec-p53 and p53-pAbAi co-transformed yeast cells; Negative control: pGADT7 and pAbAi-PPnOLP1 co-transformed yeast cells

The PnWRKY15-His recombinant protein was expressed in *Escherichia coli* and purified. The SDS-PAGE analysis indicated that the PnWRKY15-His fusion protein was approximately 36.7 kDa (Supplementary Material 3: Fig. S1 and S2). Additionally, the PPnOLP1 W-box sequence was synthesized and labeled with biotin. Electrophoretic mobility shift assay (EMSA) was conducted and it provided evidence of interaction between PnWRKY15 and the W-box of PPnOLP1 in vitro (Fig. 6A). The delayed migration of the band in lane 2 of the gel reflected the binding of the PnWRKY15-His recombinant protein to the W-box probes to form a complex with a higher molecular weight than PnWRKY15-His. The opposite result was observed in lane 4 and there were no bands that migrated more slowly in the gel than the biotin labeled free probes when the W-box was mutated.

Whether PnWRKY15 can activate the transcription of *PnOLP1* was assessed by conducting a yeast one-hybrid (Y1H) assay (Fig. 6B). The yeast cells with pAbAi-PPnOLP1/pGADT7-*PnWRKY15* were added to SD/−Leu solid medium containing 200 ng/mL ABA. These cells grew similarly to the positive control cells (i.e., Y1Hgold cells containing pAbAi-p53/pGADT7-*P53*). Conversely, the negative control yeast cells containing pAbAi-PPnOLP1/pGADT7 were unable to survive on the selection medium. These results indicated that PnWRKY15 can activate *PnOLP1* transcription in yeast.

### *PnOLP1* expression was up-regulated by PnWRKY15

**Fig. 7:**
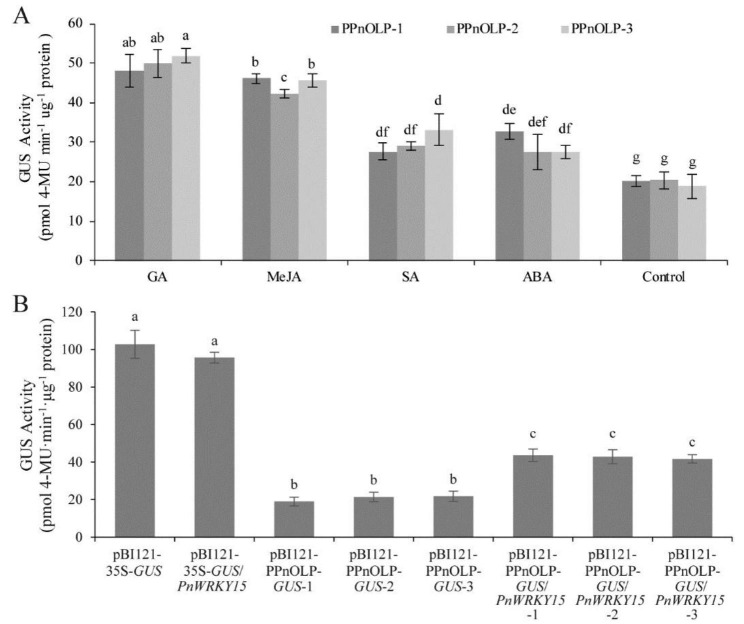
Analysis of GUS activity in transgenic tobacco. **(A)** Transcription activation of PPnOLP1 induced by plant hormone. PPnOLP-1/-2/-3: three pBI121-PPnOLP-*GUS* transgenic tobacco. **(B)** GUS activity analysis of PPnOLP1-*GUS* transgenic tobacco and PPnOLP1-*GUS*/*PnWRKY15* transgenic tobacco. pBI121-35S-*GUS*: pBI121-35S-*GUS* transgenic tobacco; pBI121-35S-*GUS*/*PnWRKY15*: pBI121-35S-*GUS*/*PnWRKY15* transgenic tobacco; pBI121-PPnOLP1-*GUS*-1/-2/-3: pBI121-PPnOLP1-*GUS* transgenic tobacco lines; pBI121-PPnOLP1-*GUS*/*PnWRKY15*-1/-2/-3: pBI121-PPnOLP1-*GUS*/*PnWRKY15* transgenic tobacco. The results were shown as average values calculated from three replicates and the significance was determined by the Student’ s *t*-test. a ~ f: significance level at *P* < 0.05

On the basis of the EMSA and Y1H assay results, the PPnOLP1-*GUS* construct was inserted into tobacco to determine the regulatory effects of PnWRKY15 on *PnOLP1* transcription in vivo. Firstly, the PPnOLP1 activity was evaluated by measuring the β-glucuronidase (GUS) activity of the transgenic tobacco carrying PPnOLP1-*GUS*. Three randomly selected PPnOLP1-*GUS* tobacco lines were treated with plant hormones, including MeJA, SA, ABA, and gibberellic acid (GA) (Fig. 7A). The GUS activity increased significantly after the MeJA treatment. More specifically, the average GUS activity after the MeJA treatment was approximately 2-times higher than the untreated control. In addition, the GUS activity in the PPnOLP1-*GUS* tobacco lines treated with ABA and SA was 27–33 pmol 4-methylumbelliferone (4-MU) min^− 1^ µg^− 1^ protein, which was higher than the control activity (18.9–20.4 pmol 4-MU min^− 1^ µg^− 1^ protein), implying that both SA and ABA can activate PPnOLP1 transcription. Thus, PPnOLP1 is an inducible promoter that is responsive to plant hormones.

The GUS activity was also measured in the PPnOLP1-*GUS*/*PnWRKY15*-OE tobacco lines (Fig. 7B). There was no significant difference in the GUS activity between the pBI121-35S-*GUS*/*PnWRKY15* tobacco and the pBI121-35S-*GUS* tobacco, indicating PnWRKY15 does not interact with the CaMV 35S promoter. GUS activity was reduced when it was fused with PPnOLP1 promoter, however, when it was co-expressed with *PnWRKY15*, the GUS activity (approximately 40 pmol 4-MU min^− 1^ µg^− 1^ protein) was about 2-times of that from the PPnOLP1-*GUS* tobacco. These results indicated that PnWRKY15 can activate the transcription of *PnOLP1*.

## Discussion

The WRKY family is one of the largest TF families in plants, with important roles in immune responses to pathogens. Specifically, WRKY TFs can directly interact with their target genes to activate or inhibit plant defense activities. Moreover, WRKY TFs, mitogen-activated protein kinases, and hormone signaling pathways cooperatively regulate immune responses [20]. In this study, a *P. notoginseng* WRKY TF (WRKY15) was identified and functionally characterized in terms of its contribution to the plant response to root rot. The subcellular localization experiment indicated PnWRKY15 is a nuclear protein. The down-regulated expression of *PnWRKY15* via RNAi in *P. notoginseng* leaves increased the susceptibility to root rot pathogen *F. solani*. In contrast, ectopic expression of *PnWRKY15* in transgenic tobacco conferred a high level of resistance to *F. solani*. Thus, PnWRKY15 positively regulates *P. notoginseng* resistance to root rot, similar to other WRKY TFs. For example, overexpression of the *Jatropha curcas WRKY2* gene in tobacco reportedly increases the resistance to *Macrophomina phaseolina* [21]. The virus-induced silencing of the *Paeonia lactiflora WRKY65* gene increases the susceptibility of plants to *Alternaria tenuissima* [22].

In response to biotic and abiotic stresses, multiple TFs, including WRKY TFs, are activated to modify the expression of defense-related genes (i.e., transcriptome reprogramming). In apple, WRKY46 enhances the resistance to *B. dothidea* by binding to the W-box element in the *MdPBS3.1* promoter and up-regulating *MdPBS3.1* expression [23]. In wild lily (*Lilium regale* Wilson) infected with *F. oxysporum*, WRKY1 positively modulates the expression of the resistance gene *LrPR10-5*, leading to strong Fusarium-wilt resistance [24]. In the current study, the expression levels of three disease resistance-related genes (*Ntosmotin*, *NtGLU1*, and *NtCHI*) were up-regulated in the *PnWRKY15*-OE tobacco lines. Additionally, *PnOLP1* expression was induced by MeJA and SA. An earlier study showed *PnOLP1* is involved in the defense response to *F. solani* [15]. The PnOLP1 recombinant protein expressed in *E. coli* has antifungal effects on several phytopathogens, including *F. solani*. Moreover, *PnOLP1*-OE tobacco lines exhibit increased resistance to *F. solani*[15]. The EMSA and Y1H assay results revealed that PnWRKY15 directly and specifically binds to the *PnOLP1* promoter and trans-activates *PnOLP1*. The subsequent co-expression of *PnOLP1* promoter and *PnWRKY15* in tobacco indicated that PnWRKY15 can activate the transcription of *PnOLP1*. These findings of this study suggest PnWRKY15 alters *PnOLP1* transcription to enhance the *P. notoginseng* defense response to root rot.

**Fig. 8:**
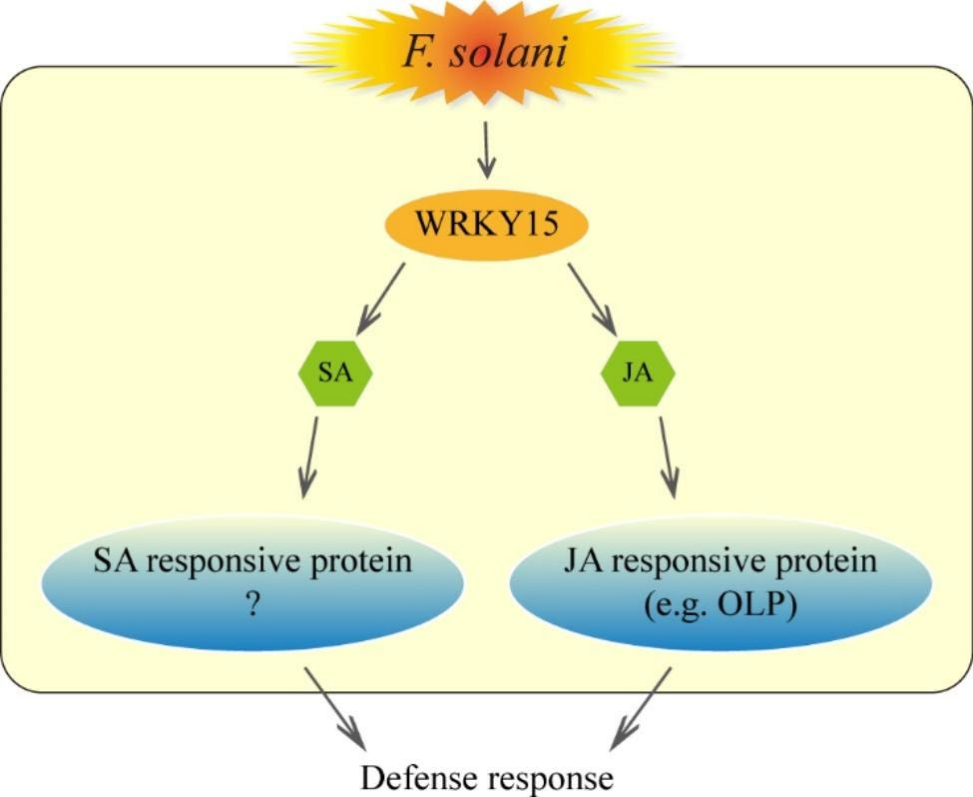
Model diagram about regulatory mechanism of PnWRKY15 in *P. notoginseng* during response to *F. salani* infection. After *P. notoginseng* was infected by the root rot pathogen, PnWRKY15 activated the JA/SA signaling pathways. Moreover, the expression of JA/SA responsive genes including the *PnOLP1* was up-regulated and then enhanced the defense response to *F. solani*

In addition to modulating the expression of many disease resistance-related genes, WRKY TFs are involved in the cross-talk between plant hormone signaling pathways under stress conditions. In cotton, GhWRKY70 has a major role in the regulation of the JA and SA signaling pathways in response to *Verticillium dahliae* infections [25]. More specifically, it negatively regulates the resistance to *V. dahliae* by activating the expression of *GhNPR1* and *GhPR1*, but inhibiting the expression of *GhPDF1.2* and *GhPR3*. In tomato (*Solanum lycopersicum*), the overexpression of *WRKY46* represses the expression of JA and SA signaling pathway marker genes, *PR1*, and protease inhibitor genes (*PI I* and *PI II*), which increases the susceptibility to *B. cinerea* [26]. In the present study, we completed an in-depth investigation of the mechanism underlying the regulatory effects of PnWRKY15 on *P. notoginseng* resistance to *F. solani*. The RNA-seq analysis showed that SA and JA signaling pathways were more active in the *PnWRKY15*-OE tobacco than that in the WT tobacco. The qRT-PCR data and hormone contents confirmed that JA/SA biosynthesis and signaling were induced by the ectopic expression of *PnWRKY15* in tobacco. Therefore, the positive regulatory effects of PnWRKY15 on JA and SA signaling pathways are important for activating the *P. notoginseng* defense response to *F. solani* (Fig. 8).

The network regulating plant immune responses, which includes WRKY TFs, is highly complex. The synergistic effects of *P. notoginseng* WRKY9 and the JA signaling pathway on the expression of an antimicrobial peptide gene (*PnDef1*) in response to *F. solani* have been reported [17]. *Ocimum sanctum* WRKY1 positively regulates the phenylpropanoid pathway, resulting in increased rosmarinic acid contents in transgenic *A. thaliana* and enhanced resistance to *Pseudomonas syringae* pv. *tomato* [27]. The ectopic expression of *WRKY31*, which was identified in an *Erysiphe necator*-resistant grape (*Vitis quinquangularis*), protects *Vitis vinifera* from *E. necator* by promoting SA signaling and inducing stilbene biosynthesis [28]. Interestingly, compared with the WT tobacco, the phenylpropanoid pathway was more active in the *PnWRKY15*-OE tobacco, but photosynthetic activities were substantially suppressed. Photosynthesis is essential for normal plant growth and development, but it is affected when plants are subjected to biotic and abiotic stresses [29]. Future studies should explore whether PnWRKY15 promotes phenylpropanoid accumulation and regulates the dynamic equilibrium between growth and defense responses following an infection by *F. solani*. Furthermore, development of a genetic transformation system for *P. notoginseng* will enable researchers to comprehensively characterize the mechanism by which WRKY TFs regulate the *P. notoginseng* defense response to root rot.

## Conclusion

In this study, silencing of *PnWRKY15* increased the sensitivity of *P. notoginseng* to the root rot pathogen *F. solani*, whereas its ectopic expression in tobacco increased the resistance to *F. solani*. The PnWRKY15 protein was localized in the nucleus and activated the transcription of the *F. solani* resistance-related gene *PnOLP1* by binding to the W-box element in the promoter. The PnWRKY15 TF positively modulated *PnOLP1* expression and induced the SA and JA signaling pathways as well, during the defense response to *F. solani*. The data generated in this study indicate that PnWRKY15 is a TF that enhances root rot resistance by up-regulating *PnOLP1* expression and inducing JA/SA signaling pathways.

## Materials and methods

### Plant and fungal materials

The 1-year-old *P. notoginseng* plants and wild-type tobacco (*Nicotiana tabacum* L.) seeds were provided by Dr. Guanze Liu from Yunnan Agricultural University, Kunming, China. The young leaves of *P. notoginseng* plants grown under a shade net were used for the cloning of *PnWRKY15* and PPnOLP1 as well as the RNAi experiment. *Fusarium solani* was preserved in our laboratory and activated on potato dextrose agar medium before use. The wild-type tobacco seeds were surface-sterilized in 75% alcohol and a 0.1% HgCl_2_ solution before they were sown on quarter-strength Murashige and Skoog (MS) medium. Two-month-old seedlings were used for the genetic transformation.

### Cloning of *PnWRKY15*

Gene-specific primers (Supplementary Material 1: Table S4) were designed on the basis of the *PnWRKY15* cDNA sequence obtained from [17] to amplify the *PnWRKY15* ORF via RT-PCR using the Eastep® RT Master Mix kit (Promega, USA). The PCR product was sequenced after T-A cloning to verify that the *PnWRKY15* sequence was correct. The conserved domain in the PnWRKY15 amino acid sequence was used to search the NCBI database (https://www.ncbi.nlm.nih.gov/guide/homology/). The PnWRKY15 amino acid sequence homology was analyzed using the tblast online tool (https://blast.ncbi.nlm.nih.gov/Blast.cgi).

### Subcellular localization

The *PnWRKY15* ORF was ligated into the pBINm-gfp5 vector digested with *Bam*HI and *Xba*I to generate the *PnWRKY15-GFP* construct (Supplementary Material 1: Table S4). The recombinant vector was transferred into *Agrobacterium tumefaciens* EHA105 cells using liquid nitrogen (i.e., quick freezing-based method). Onion epidermal cells were transformed with *A. tumefaciens* containing pBINm-gfp5-*PnWRKY15* or the pBINm-gfp5 empty vector and then cultured on MS medium for 2 days in darkness before examining the localization of green fluorescence using a laser scanning confocal microscope (Nikon, Japan). The red fluorescence of propidium iodide was observed to indicate the nucleus in the onion epidermal cells.

### RNAi

Gene-specific primers with attB linkers were designed (Supplementary Material 1: Table S4) for the PCR amplification of a *PnWRKY15* fragment (420 bp) fused with attB linkers. The pHellsgate 2-*PnWRKY15* vector was constructed using the Gateway BP Clonase™ II Enzyme Mix kit (Invitrogen, USA) and was then transformed into *A. tumefaciens* EHA105 cells. Young leaves from 1-year-old *P. notoginseng* plants grown under normal conditions were wounded using a sterile syringe and then divided into two groups. The *A. tumefaciens* cells containing pHellsgate 2*-PnWRKY15* were added to the wounded leaves in one group, whereas the *A. tumefaciens* cells containing the pHellsgate 2 empty vector were added to the wounded leaves in the other group. The leaves in both groups were placed on moistened sterile filter paper and incubated for 24 h at 25 °C in darkness. All of the leaves were inoculated with *F. solani* spore suspensions and incubated for 72 h under light. The disease symptoms were recorded using a digital camera (Nikon). The lesion areas were calculated using Adobe Photoshop 2020. The *PnWRKY15*, *PnAOS*, *PnPR-1*, *PnMYC2*, *PnCHI*, and *PnOLP1* expression levels in the leaves were analyzed by qRT-PCR using gene-specific primers (Supplementary Material 1: Table S5).

### Development of *PnWRKY15*-OE tobacco lines and analysis of disease resistance

The pCAMBIA2300S-*PnWRKY15* vector was constructed as described above (primer sequences are listed in Supplementary Material 1: Table S4) and then transferred into WT tobacco leaf discs via *A. tumefaciens* LBA4404. The leaf discs were induced to produce adventitious buds in MS medium containing kanamycin, 1-naphthylacetic acid, and cytokinin. The buds were transferred to half-strength MS medium containing kanamycin to induce rooting under light (26 °C, 16-h light:8-h dark cycle). Genomic DNA was extracted from the regenerated tobacco seedlings using cetyltrimethylammonium bromide (CTAB) to identify transgenic plants. The confirmed transgenic seedlings were transferred to the greenhouse. The T_2_ lines used in the subsequent experiments were obtained through self-pollination.

Selected T_2_*PnWRKY15*-OE tobacco lines were used for a qRT-PCR analysis of *PnWRKY15* expression levels using the Eastep® qPCR Master Mix kit (Promega), with WT tobacco serving as the negative control (primer sequences are listed in Supplementary Material 1: Table S5). The tobacco *Ntactin* gene (AB158612.1) was chosen as the internal reference to calculate relative gene expression levels according to the 2^−ΔΔCt^ method. This experiment included three biological replicates. The generated data were analyzed by performing *t*-tests.

The four *PnWRKY15*-OE tobacco lines with high *PnWRKY15* expression levels were analyzed regarding their resistance to *F. solani*. The *F. solani* spore suspension was prepared after activating the fungus on potato dextrose agar medium. Young tobacco leaves were harvested and wounded using a sterile pipette tip before they were inoculated with the spore suspension. The inoculated leaves were placed on moistened filter paper and incubated for 7 days under light (26 °C, 16-h light:8-h dark cycle). Tobacco roots were immersed in an *F. solani* spore suspension for 30 min. The inoculated tobacco plants were grown under hydroponic conditions in an illumination incubator for 7 days. The disease symptoms were recorded using a digital camera and the lesion areas were calculated.

### Transcriptome sequencing analysis of the *PnWRKY15*-OE tobacco

Total RNA was extracted from the young leaves of WT and *PnWRKY15*-OE tobacco (line 15 - 2) plants grown under normal conditions. After determination of the RNA concentration, purity, and integrity using the NanoDrop 2000 spectrophotometer (Thermo Fisher Scientific, USA) and the Agilent Bioanalyzer 2100 (Agilent Technologies Inc., USA), the high-quality RNA was used to construct the sequencing library, which was analyzed using the Illumina NovaSeq 6000 sequencing platform. The RNA-seq analysis was performed by Shanghai Majorbio Bio-pharm Technology Co., Ltd. The transcriptome quality assessment, transcriptome assembly, analysis of the correlation between samples, analysis of the DEGs, gene ontology functional annotation, and KEGG pathway analysis [30–32] were performed using the Majorbio Cloud Platform (www.majorbio.com). The RNA-seq experiment involved three biological replicates.

To verify the DEGs identified by the RNA-seq analysis, the relative expression levels of some genes related to JA biosynthesis and JA/SA signal transduction as well as a few PR genes were determined by qRT-PCR. This analysis was completed using line 15 - 2 (i.e., the line used for the RNA-seq analysis) and two randomly selected tobacco lines (15-21/-24). The qRT-PCR assay was performed as described above using gene-specific primers (Supplementary Material 1: Table S5).

### Hormone contents determination of *PnWRKY15*-OE tobacco lines

An ultra-high performance liquid chromatography-mass spectrometry system was used to measure the JA and SA contents in the *PnWRKY15*-OE tobacco lines (15 − 2/-21/-24) and the WT tobacco as previously described [33]. The chromatographic peak area for the JA or SA in each sample and the linear equation of the standard curve were used to calculate the corresponding concentrations in the tested samples.

### Cloning of PPnOLP1

Genomic DNA was extracted from young *P. notoginseng* leaves using the CTAB method. Two nested PCR primers (*PnOLP1*-GSP1 and *PnOLP1*-GSP2, Supplementary Material 1: Table S4) were designed to amplify the promoter of *PnOLP1*, which encodes an osmotin-like protein that confers resistance to *F. solani* [15]. The PPnOLP1 sequence was cloned according to the Universal GenomeWalker 2.0 user manual (Takara, Japan). The *cis*-elements in PPnOLP1 were identified using PLANTCARE (http://bioinformatics.psb.ugent.be/webtools/plantcare/html/).

### EMSA

Gene-specific primers were designed and used to amplify *PnWRKY15* (without the stop codon) (Supplementary Material 1: Table S4). The pET32a-*PnWRKY15* recombinant vector was constructed using the ClonExpress II One Step Cloning Kit (Vazyme, China) and then inserted into *E. coli* BL21 (DE3) cells (Tsingke Biotechnology, China). The expression of the recombinant PnWRKY15 protein was induced by adding 2 mM isopropyl-β-D-1-thiogalactoside to the bacterial solution and incubating at 25 °C for 8 h. The protein was denatured and purified as previously described [24]. The PPnOLP1 sequence was used to design the following probes containing the W-box element: biotin labeled probe, unlabeled competitor probe, and biotin labeled mutant probe (i.e., mutated W-box) (Supplementary Material 1: Table S4). The probes were synthesized and labeled with biotin (Sangon Biotech, China), except for the unlabeled competitor probe. The EMSA was performed using the LightShift™ Chemiluminescent EMSA kit (Pierce, USA).

### Y1H assay

A pair of gene-specific primers (Supplementary Material 1: Table S5) was designed to construct the pAbAi-PPnOLP1 decoy vector (Clontech, USA). The pAbAi-PPnOLP1 recombinant vector was incorporated into Y1Hgold yeast cells, which were then grown on SD/−Ura medium. The pGADT7-*PnWRKY15* prey vector (Clontech) was constructed after the *PnWRKY15* ORF was amplified by PCR using gene-specific primers (Supplementary Material 1: Table S4). The prey vector was inserted into Y1Hgold yeast cells, which were grown on SD/−Leu medium. The pAbAi-p53 decoy vector and the pGADT7-*P53* prey vector from the Matchmaker® Gold Yeast One-Hybrid Library Screening System Kit (Clontech) were used as positive controls. The Y1Hgold yeast cells carrying pAbAi-p53/pGADT7-*P53* (positive control), pAbAi-PPnOLP1/pGADT7 AD (negative control), and pAbAi-PPnOLP1/pGADT7-*PnWRKY15* were added to plates containing SD/−Leu/AbA medium supplemented with 200 ng/mL ABA. The plates were incubated at 28 °C for 3 days to confirm whether PnWRKY15 can interact with PPnOLP1.

### Analysis of GUS activity

The pBI121-PPnOLP1-*GUS* recombinant vector was constructed and then inserted into *A. tumefaciens* LBA4404 cells for the transformation of WT tobacco and *PnWRKY15*-OE tobacco. The tobacco shoots were induced in a tissue culture system. The PPnOLP1-*GUS* and PPnOLP1-*GUS*/*PnWRKY15* transgenic tobacco plants were verified by PCR analysis. Leaf discs from three PPnOLP1-*GUS* transgenic tobacco plants were treated with sterile water (control), 100 µM MeJA, 100 µM SA, 100 µM ABA, or 100 µM GA solutions for 2 h. The GUS activity was measured using a fluorescence spectrophotometer as previously described [34]. The GUS activity of the PPnOLP1-*GUS*/*PnWRKY15* transgenic tobacco was determined using pBI121-*GUS* transgenic tobacco as the control. The GUS activity assay included three biological replicates.

### Data analyses

The gene expression, lesion area, GUS activity, and JA/SA content data were analyzed using Excel 2019 and SPSS 23.0.

## Electronic supplementary material

Below is the link to the electronic supplementary material.

Supplementary Material 1

Supplementary Material 2

Supplementary Material 3

Supplementary Material 4

## Data Availability

The original contributions presented in the study are publicly available. The PPnOLP1 data generated in this study have submitted to NCBI GenBank database (https://www.ncbi.nlm.nih.gov/, and the GenBank accession number is OP970657).
