# Supplementary material for: Panax notoginseng transcription factor WRKY15 modulates resistance to Fusarium solani by up-regulating osmotin-like protein expression and inducing JA/SA signaling pathways
Source: BMC Plant Biol. 2023 Jul 17;23:362. doi: 10.1186/s12870-023-04373-x (PMC10351173; doi:10.1186/s12870-023-04373-x)
Supplement: Supplementary file 1 — Supplementary Material 1 [file 12870_2023_4373_MOESM1_ESM.docx]

Table S1 KEGG enrichment information

| Gene Num | Pathway id | Description | First Category | Second Category |
| --- | --- | --- | --- | --- |
| 115 | map04075 | Plant hormone signal transduction | Environmental Information Processing | Signal transduction |
| 72 | map00940 | Phenylpropanoid biosynthesis | Metabolism | Biosynthesis of other secondary metabolites |
| 51 | map00195 | Photosynthesis | Metabolism | Energy metabolism |
| 46 | map00196 | Photosynthesis - antenna proteins | Metabolism | Energy metabolism |
| 46 | map00040 | Pentose and glucuronate interconversions | Metabolism | Carbohydrate metabolism |
| 46 | map04626 | Plant-pathogen interaction | Organismal Systems | Environmental adaptation |
| 45 | map04016 | MAPK signaling pathway - plant | Environmental Information Processing | Signal transduction |
| 44 | map00520 | Amino sugar and nucleotide sugar metabolism | Metabolism | Carbohydrate metabolism |
| 44 | map00500 | Starch and sucrose metabolism | Metabolism | Carbohydrate metabolism |
| 37 | map00564 | Glycerophospholipid metabolism | Metabolism | Lipid metabolism |
| 36 | map04144 | Endocytosis | Cellular Processes | Transport andcatabolism |
| 35 | map00270 | Cysteine and methionine metabolism | Metabolism | Amino acid metabolism |
| 34 | map00480 | Glutathione metabolism | Metabolism | Metabolism of other amino acids |
| 34 | map00630 | Glyoxylate and dicarboxylate metabolism | Metabolism | Carbohydrate metabolism |
| 32 | map00860 | Porphyrin and chlorophyll metabolism | Metabolism | Metabolism of cofactors and vitamins |
| 31 | map03030 | DNA replication | Genetic Information Processing | Replication and repair |
| 31 | map04141 | Protein processing in endoplasmic reticulum | Genetic Information Processing | Folding, sorting and degradation |
| 30 | map00592 | alpha-Linolenic acid metabolism | Metabolism | Lipid metabolism |
| 30 | map03010 | Ribosome | Genetic Information Processing | Translation |
| 29 | map00010 | Glycolysis / Gluconeogenesis | Metabolism | Carbohydrate metabolism |

Table S2 The information of gene heat map

| Gene_id | Control | PnWRKY15 | Gene description |
| --- | --- | --- | --- |
| gene_77178 | 0.59 | 6.37 | OPC-8:0 CoA ligase1 |
| gene_6774 | 10.09 | 25.33 | Oxidoreductase, zinc-binding dehydrogenase family protein |
| gene_76378 | 15.30 | 37.00 | Oxidoreductase, zinc-binding dehydrogenase family protein |
| gene_29638 | 0.51 | 1.55 | acyl-CoA oxidase 2 |
| gene_4742 | 3.46 | 9.76 | Auxin-responsive GH3 family protein |
| gene_6599 | 74.99 | 42.28 | jasmonate-zim-domain protein 3 |
| gene_24945 | 8.09 | 2.30 | TIFY domain/Divergent CCT motif family protein |
| gene_48999 | 32.59 | 14.71 | TIFY domain/Divergent CCT motif family protein |
| gene_80914 | 172.52 | 797.64 | Copper amine oxidase family protein |
| gene_80912 | 126.75 | 686.80 | copper amine oxidase family protein |
| gene_43960 | 89.68 | 314.49 | Copper amine oxidase family protein |
| gene_53065 | 58.55 | 194.98 | copper amine oxidase family protein |
| gene_66573 | 2.63 | 7.47 | 3-hydroxyacyl-CoA dehydrogenase family protein |
| gene_59578 | 0.72 | 2.82 | tryptophan synthase alpha chain |
| gene_76084 | 5.44 | 15.11 | bZIP transcription factor family protein |
| gene_1987 | 4.76 | 11.46 | bZIP transcription factor family protein |
| gene_65035 | 0.32 | 0.92 | bZIP transcription factor family protein |
| gene_53862 | 489.87 | 1144.62 | Papain family cysteine protease |
| gene_71663 | 4.39 | 12.79 | Leucine-rich repeat protein kinase family protein |
| gene_82719 | 8.30 | 19.04 | Leucine-rich repeat protein kinase family protein |
| gene_13066 | 1.30 | 4.14 | EF-TU receptor |
| gene_10032 | 1.10 | 3.90 | Protein kinase superfamily protein |
| gene_50901 | 1.99 | 4.98 | Protein kinase superfamily protein |
| gene_24577 | 0.95 | 2.45 | chitin elicitor receptor kinase 1 |
| gene_59894 | 0.69 | 2.05 | chitin elicitor receptor kinase 1 |
| gene_17082 | 2.17 | 6.95 | calmodulin 7 |
| gene_18199 | 1.28 | 3.29 | Protein kinase superfamily protein |
| gene_30963 | 0.43 | 1.05 | Leucine-rich repeat protein kinase family protein |
| gene_39203 | 9.68 | 3.54 | 3-ketoacyl-CoA synthase 1 |
| gene_26163 | 72.73 | 37.33 | 3-ketoacyl-CoA synthase 2 |
| gene_26336 | 6.62 | 1.95 | 3-ketoacyl-CoA synthase 6 |
| gene_3684 | 45.98 | 22.46 | 3-ketoacyl-CoA synthase 6 |
| gene_64031 | 29.27 | 5.01 | 3-ketoacyl-CoA synthase 2 |
| gene_38320 | 7.86 | 2.95 | 3-ketoacyl-CoA synthase 6 |
| gene_69519 | 4.87 | 1.46 | 3-ketoacyl-CoA synthase 1 |
| gene_63161 | 8.99 | 4.91 | 3-ketoacyl-CoA synthase 6 |
| gene_69278 | 8.54 | 4.45 | 3-ketoacyl-CoA synthase 6 |
| gene_62471 | 0.35 | 0.03 | 3-ketoacyl-CoA synthase 11 |
| gene_21222 | 3.96 | 12.52 | NB-ARC domain-containing disease resistance protein |
| gene_30516 | 5.78 | 19.26 | NB-ARC domain-containing disease resistance protein |
| gene_8227 | 1.43 | 4.49 | NB-ARC domain-containing disease resistance protein |
| gene_57536 | 0.45 | 1.17 | NB-ARC domain-containing disease resistance protein |
| gene_19970 | 0.49 | 1.40 | NB-ARC domain-containing disease resistance protein |
| gene_57858 | 0.94 | 2.48 | NB-ARC domain-containing disease resistance protein |
| gene_41446 | 1.74 | 4.74 | NB-ARC domain-containing disease resistance protein |
| gene_13631 | 0.22 | 0.58 | NB-ARC domain-containing disease resistance protein |
| gene_28794 | 2.49 | 8.94 | disease resistance protein (TIR-NBS-LRR class), putative |
| gene_20114 | 1.32 | 4.44 | disease resistance protein (TIR-NBS-LRR class), putative |
| gene_49971 | 0.86 | 2.08 | disease resistance protein (TIR-NBS-LRR class), putative |
| gene_75003 | 0.43 | 0.86 | disease resistance protein (TIR-NBS-LRR class), putative |
| gene_25394 | 2.09 | 22.59 | Disease resistance protein (TIR-NBS-LRR class) family |
| gene_42851 | 0.58 | 1.67 | Disease resistance protein (TIR-NBS-LRR class) family |
| gene_16863 | 0.23 | 1.45 | Disease resistance protein (TIR-NBS-LRR class) family |
| gene_7787 | 1.58 | 3.90 | Disease resistance protein (CC-NBS-LRR class) family |
| gene_57893 | 0.71 | 1.71 | Disease resistance protein (CC-NBS-LRR class) family |
| gene_1939 | 1.26 | 3.35 | Disease resistance protein (CC-NBS-LRR class) family |
| gene_29303 | 0.27 | 0.88 | Disease resistance protein (CC-NBS-LRR class) family |
| gene_40547 | 0.21 | 0.59 | Disease resistance protein (CC-NBS-LRR class) family |
| gene_84150 | 3.65 | 10.86 | camphor resistance CrcB family protein |
| gene_84724 | 3.42 | 12.27 | pathogenesis-related family protein |
| gene_36979 | 6.08 | 20.05 | plant intracellular ras group-related LRR 4 |
| gene_441 | 3.06 | 8.52 | Leucine-rich repeat (LRR) family protein |
| gene_60594 | 0.53 | 1.62 | Pentatricopeptide repeat (PPR) superfamily protein |
| gene_73283 | 99.84 | 275.20 | Pentatricopeptide repeat (PPR-like) superfamily protein |
| gene_65545 | 5.55 | 13.80 | cellulose synthase like E1 |
| gene_54659 | 21.77 | 56.94 | cellulose synthase like G1 |
| gene_45651 | 0.02 | 0.74 | cellulose synthase like G2 |
| gene_67147 | 26.89 | 67.63 | cytochrome P450, family 94, subfamily D, polypeptide 1 |
| gene_71142 | 57.97 | 256.72 | cytochrome P450, family 83, subfamily B, polypeptide 1 |
| gene_9080 | 1.33 | 5.44 | cytochrome P450, family 83, subfamily B, polypeptide 1 |
| gene_50700 | 0.99 | 4.94 | cytochrome P450, family 82, subfamily G, polypeptide 1 |
| gene_42339 | 0.65 | 2.84 | cytochrome P450, family 82, subfamily C, polypeptide 4 |
| gene_27235 | 0.47 | 1.87 | cytochrome P450, family 82, subfamily C, polypeptide 4 |
| gene_27634 | 1.13 | 3.60 | cytochrome P450, family 82, subfamily C, polypeptide 4 |
| gene_64246 | 4.04 | 20.85 | cytochrome P450, family 81, subfamily K, polypeptide 2 |
| gene_78175 | 2.06 | 6.57 | cytochrome P450, family 81, subfamily K, polypeptide 2 |
| gene_24468 | 2.72 | 6.79 | cytochrome P450, family 81, subfamily D, polypeptide 4 |
| gene_8287 | 11.98 | 28.18 | cytochrome P450, family 78, subfamily A, polypeptide 6 |
| gene_6084 | 1.24 | 10.64 | cytochrome P450, family 76, subfamily G, polypeptide 1 |
| gene_37144 | 1.16 | 3.23 | cytochrome P450, family 76, subfamily C, polypeptide 2 |
| gene_56003 | 1.34 | 3.07 | cytochrome P450, family 722, subfamily A, polypeptide 1 |
| gene_65241 | 0.00 | 0.22 | cytochrome P450, family 721, subfamily A, polypeptide 1 |
| gene_73505 | 8.14 | 31.04 | cytochrome P450, family 72, subfamily A, polypeptide 15 |
| gene_34094 | 12.30 | 34.50 | cytochrome P450, family 72, subfamily A, polypeptide 15 |
| gene_10376 | 15.69 | 38.62 | cytochrome P450, family 72, subfamily A, polypeptide 15 |
| gene_27604 | 0.73 | 2.02 | cytochrome P450, family 72, subfamily A, polypeptide 15 |
| gene_58336 | 0.42 | 1.46 | cytochrome P450, family 72, subfamily A, polypeptide 11 |
| gene_31288 | 5.26 | 30.63 | cytochrome P450, family 716, subfamily A, polypeptide 1 |
| gene_37825 | 8.23 | 26.38 | cytochrome P450, family 71, subfamily B, polypeptide 36 |
| gene_69030 | 16.00 | 68.15 | cytochrome P450, family 71, subfamily B, polypeptide 35 |
| gene_77192 | 1.47 | 5.33 | cytochrome P450, family 71, subfamily B, polypeptide 35 |
| gene_38084 | 5.83 | 24.13 | cytochrome P450, family 71, subfamily B, polypeptide 35 |
| gene_37883 | 2.41 | 8.27 | cytochrome P450, family 71, subfamily B, polypeptide 34 |
| gene_21673 | 3.24 | 10.44 | cytochrome P450, family 71, subfamily B, polypeptide 34 |
| gene_55282 | 1.62 | 5.80 | cytochrome P450, family 71, subfamily B, polypeptide 34 |
| gene_79737 | 2.55 | 10.57 | cytochrome P450, family 71, subfamily B, polypeptide 34 |
| gene_17920 | 16.31 | 42.48 | cytochrome P450, family 71, subfamily A, polypeptide 25 |
| gene_777 | 1.05 | 3.16 | cytochrome P450, family 71, subfamily A, polypeptide 25 |
| gene_58009 | 0.48 | 4.82 | cytochrome P450, family 704, subfamily A, polypeptide 2 |
| gene_3434 | 1.07 | 11.43 | Cytochrome P450 superfamily protein |
| gene_70250 | 3.13 | 8.58 | Cytochrome P450 superfamily protein |
| gene_47717 | 1.07 | 10.28 | Cytochrome P450 superfamily protein |
| gene_42390 | 0.53 | 1.31 | cytochrome p450 81d1 |
| gene_41822 | 0.78 | 2.71 | 2-oxoglutarate (2OG) and Fe(II)-dependent oxygenase superfamily protein |
| gene_53947 | 0.10 | 2.20 | beta glucosidase 17 |
| gene_3217 | 0.35 | 1.10 | cinnamyl alcohol dehydrogenase 9 |
| gene_70068 | 2.93 | 7.35 | HXXXD-type acyl-transferase family protein |
| gene_47769 | 1.64 | 10.53 | peroxidase 2 |
| gene_14429 | 0.05 | 0.53 | Peroxidase superfamily protein |
| gene_29358 | 1.96 | 8.88 | Peroxidase superfamily protein |
| gene_58827 | 1.29 | 7.43 | Peroxidase superfamily protein |
| gene_55552 | 0.13 | 0.75 | Peroxidase superfamily protein |
| gene_62123 | 2.18 | 9.20 | phenylalanine ammonia-lyase 2 |
| gene_74391 | 16.05 | 9.07 | beta glucosidase 17 |
| gene_75692 | 2.18 | 0.26 | beta glucosidase 17 |
| gene_49249 | 8.85 | 2.96 | beta glucosidase 40 |
| gene_34222 | 7.46 | 3.83 | beta glucosidase 40 |
| gene_73584 | 0.47 | 0.01 | beta-glucosidase 47 |
| gene_34944 | 67.63 | 30.95 | B-S glucosidase 44 |
| gene_79577 | 56.05 | 31.33 | B-S glucosidase 44 |
| gene_83556 | 34.92 | 10.84 | cinnamyl alcohol dehydrogenase 9 |
| gene_27391 | 3.42 | 0.29 | FAD-binding Berberine family protein |
| gene_26685 | 3.07 | 0.30 | FAD-binding Berberine family protein |
| gene_9342 | 2.47 | 0.38 | Glycosyl hydrolase family protein |
| gene_26330 | 32.52 | 18.65 | Glycosyl hydrolase family protein |
| gene_68835 | 3.06 | 0.42 | GroES-like zinc-binding alcohol dehydrogenase family protein |
| gene_60014 | 15.96 | 8.61 | HXXXD-type acyl-transferase family protein |
| gene_56641 | 12.36 | 6.09 | HXXXD-type acyl-transferase family protein |
| gene_11059 | 15.37 | 8.83 | nascent polypeptide-associated complex subunit alpha-like protein 2 |
| gene_61589 | 8.83 | 4.94 | O-methyltransferase 1 |
| gene_18630 | 316.24 | 105.49 | peroxidase 2 |
| gene_82073 | 2.51 | 0.86 | Peroxidase superfamily protein |
| gene_80730 | 25.22 | 10.74 | Peroxidase superfamily protein |
| gene_66387 | 3.85 | 0.76 | Peroxidase superfamily protein |
| gene_79396 | 17.66 | 8.80 | Peroxidase superfamily protein |
| gene_76948 | 4.18 | 1.05 | Peroxidase superfamily protein |
| gene_39154 | 1.89 | 0.09 | Peroxidase superfamily protein |
| gene_9714 | 2.40 | 0.21 | Peroxidase superfamily protein |
| gene_80729 | 1.13 | 0.00 | Peroxidase superfamily protein |
| gene_22353 | 2.64 | 0.70 | Peroxidase superfamily protein |
| gene_80731 | 3.18 | 0.74 | Peroxidase superfamily protein |
| gene_20009 | 3.45 | 1.31 | Peroxidase superfamily protein |
| gene_78170 | 2.75 | 0.49 | Peroxidase superfamily protein |
| gene_84095 | 3.42 | 1.42 | Peroxidase superfamily protein |
| gene_1446 | 2.11 | 0.51 | Peroxidase superfamily protein |
| gene_79349 | 1.72 | 0.49 | Peroxidase superfamily protein |
| gene_43528 | 2.50 | 1.44 | Peroxidase superfamily protein |
| gene_71463 | 4.80 | 2.45 | Peroxidase superfamily protein |
| gene_69241 | 0.91 | 0.14 | Peroxidase superfamily protein |
| gene_77958 | 0.69 | 0.23 | Peroxidase superfamily protein |
| gene_40864 | 245.01 | 111.52 | Peroxidase superfamily protein |
| gene_29448 | 0.53 | 0.02 | Peroxidase superfamily protein |
| gene_52982 | 1.19 | 0.05 | Peroxidase superfamily protein |
| gene_19820 | 362.26 | 163.49 | Peroxidase superfamily protein |
| gene_79404 | 8.18 | 3.82 | Peroxidase superfamily protein |
| gene_26748 | 0.62 | 0.08 | Peroxidase superfamily protein |
| gene_29449 | 0.57 | 0.12 | Peroxidase superfamily protein |
| gene_75196 | 2.57 | 0.97 | phenylalanine ammonia-lyase 2 |
| gene_75046 | 2.61 | 1.27 | phenylalanine ammonia-lyase 2 |
| gene_76210 | 1.87 | 0.98 | phenylalanine ammonia-lyase 2 |
| gene_62798 | 10.79 | 5.71 | root hair specific 19 |
| gene_20021 | 16.62 | 7.80 | S-adenosyl-L-methionine-dependent methyltransferases superfamily protein |
| gene_77000 | 1.11 | 0.42 | SNF7 family protein |
| gene_19200 | 0.83 | 0.32 | SNF7 family protein |
| gene_11368 | 732.11 | 404.26 | Chlorophyll A-B binding family protein |
| gene_51850 | 645.56 | 296.14 | photosystem I reaction center subunit PSI-N, chloroplast, putative / PSI-N, putative (PSAN) |
| gene_22727 | 594.78 | 343.47 | photosystem I reaction center subunit PSI-N, chloroplast, putative / PSI-N, putative (PSAN) |
| gene_78579 | 905.75 | 470.27 | photosystem I subunit E-2 |
| gene_46518 | 1050.32 | 505.09 | photosystem I subunit F |
| gene_53880 | 713.44 | 388.85 | photosystem I subunit F |
| gene_74250 | 137.92 | 53.27 | photosystem I subunit G |
| gene_78139 | 99.45 | 40.19 | photosystem I subunit H-1 |
| gene_2999 | 448.87 | 203.89 | photosystem I subunit H-1 |
| gene_43545 | 1031.84 | 508.61 | photosystem I subunit H-1 |
| gene_79012 | 173.36 | 48.68 | photosystem I subunit H2 |
| gene_59136 | 651.44 | 283.13 | photosystem I subunit H2 |
| gene_80940 | 948.65 | 355.50 | photosystem I subunit K |
| gene_67287 | 768.26 | 264.40 | photosystem I subunit K |
| gene_65015 | 573.72 | 265.81 | photosystem I subunit K |
| gene_328 | 569.59 | 267.10 | photosystem I subunit K |
| gene_17581 | 359.80 | 141.59 | photosystem I subunit O |
| gene_62150 | 980.35 | 341.18 | photosystem I subunit O |
| gene_82155 | 1063.77 | 488.69 | photosystem I subunit O |
| gene_17490 | 139.42 | 53.94 | photosystem II reaction center PSB28 protein |
| gene_84556 | 125.93 | 62.92 | photosystem II reaction center PSB28 protein |
| gene_30863 | 1132.39 | 537.42 | photosystem II reaction center W |
| gene_34967 | 1482.41 | 718.62 | photosystem II reaction center W |
| gene_29908 | 295.53 | 161.49 | photosystem II reaction center W |
| gene_14491 | 44.39 | 16.89 | photosystem II reaction center W |
| gene_35277 | 1023.83 | 495.38 | photosystem II subunit O-2 |
| gene_2398 | 1306.35 | 678.13 | photosystem II subunit O-2 |
| gene_36597 | 535.37 | 299.65 | photosystem II subunit O-2 |
| gene_78629 | 16.86 | 9.49 | photosystem II subunit O-2 |
| gene_75381 | 754.10 | 295.51 | photosystem II subunit P-1 |
| gene_61812 | 629.04 | 224.36 | photosystem II subunit P-1 |
| gene_68589 | 718.98 | 288.19 | photosystem II subunit P-1 |
| gene_10255 | 372.12 | 161.48 | photosystem II subunit P-1 |
| gene_2113 | 1682.50 | 779.37 | photosystem II subunit Q-2 |
| gene_71328 | 1437.87 | 718.36 | photosystem II subunit Q-2 |
| gene_61520 | 1159.41 | 299.38 | plastocyanin 1 |
| gene_18443 | 256.32 | 109.54 | plastocyanin 1 |
| gene_13929 | 242.54 | 110.07 | PS II oxygen-evolving complex 1 |
| gene_21116 | 73.64 | 33.02 | PsbQ-like 2 |
| gene_32307 | 80.60 | 43.09 | PsbQ-like 2 |
| gene_16097 | 1583.01 | 729.48 | chlorophyll A/B binding protein 1 |
| gene_16089 | 2104.65 | 1075.89 | chlorophyll A/B binding protein 1 |
| gene_69686 | 1850.36 | 962.04 | light harvesting complex of photosystem II 5 |
| gene_78936 | 2130.14 | 1085.59 | light harvesting complex photosystem II |
| gene_25091 | 2019.40 | 1056.10 | light harvesting complex photosystem II |
| gene_9305 | 372.60 | 126.63 | light harvesting complex photosystem II subunit 6 |
| gene_41304 | 404.19 | 123.28 | light harvesting complex photosystem II subunit 6 |
| gene_67730 | 240.74 | 96.26 | light-harvesting chlorophyll B-binding protein 3 |
| gene_84740 | 487.30 | 218.60 | light-harvesting chlorophyll B-binding protein 3 |
| gene_78623 | 272.20 | 137.71 | light-harvesting chlorophyll B-binding protein 3 |
| gene_18030 | 1279.92 | 588.10 | light-harvesting chlorophyll-protein complex I subunit A4 |
| gene_31680 | 94.04 | 6.51 | light-harvesting chlorophyll-protein complex I subunit A4 |
| gene_62863 | 195.37 | 96.72 | light-harvesting chlorophyll-protein complex I subunit A4 |
| gene_5524 | 338.11 | 153.19 | light-harvesting chlorophyll-protein complex I subunit A4 |
| gene_59493 | 31.71 | 9.31 | phosphate transporter 3 |
| gene_57590 | 2261.55 | 1249.97 | photosystem I light harvesting complex gene 2 |
| gene_16712 | 393.12 | 205.04 | photosystem I light harvesting complex gene 3 |
| gene_19722 | 40.74 | 18.33 | photosystem I light harvesting complex gene 5 |
| gene_35859 | 66.16 | 33.18 | photosystem I light harvesting complex gene 5 |
| gene_44436 | 803.55 | 252.73 | photosystem II light harvesting complex gene 2.1 |
| gene_28758 | 739.53 | 352.38 | photosystem II light harvesting complex gene 2.1 |
| gene_84662 | 286.83 | 105.45 | photosystem II light harvesting complex gene 2.1 |
| gene_12366 | 404.28 | 109.50 | photosystem II light harvesting complex gene 2.1 |
| gene_57594 | 14.24 | 7.20 | serine carboxypeptidase-like 42 |
| gene_9321 | 3.99 | 1.68 | Thioesterase superfamily protein |

Table S3 The *cis*-elements in the PPnOLP1

| The *cis*-element name | Sequence | Element function | Position(bp) |
| --- | --- | --- | --- |
| W-box | TTGACC | ET、SA、MeJA responsive element | -32~37 |
| CAAT-box | CAAAT | common cis-acting element in promoter and enhancer regions | -61~65  -269~273  -386~390  -869~873  -893~897 |
| Box 4 | ATTAAT | part of a conserved DNA module involved in light responsiveness | -85~90 |
| AUXRRCORE | GGTCCAT | cis-acting regulatory element involved in auxin responsiveness | -182~188 |
| LAMPELEMENT | CCTTATCCA | light responsive element | -318~326 |
| MYC | CATTTG | MYC binding site | -338~343 |
| TATA-box | TATATA | common cis-acting element in promoter and enhancer regions | -407~412 |
| ABRELATERD1 | ACGTG | *cis*-element involved in the abscisic acid responsiveness | -506~510 |
| MBSI | TAAACGTAAAAA | MYB binding site involved in flavonoid biosynthetic genes regulation | -716~727 |
| MYB | CAACAG | MYB binding site | -779~784 |
| Gap-box | CAAATGAA | part of a light responsive element | -869~876 |

Table S4 The primers used for *PnWRKY15* functional analysis

| Target genes | Primer sequences | Assays |
| --- | --- | --- |
| *PnWRKY15* | Forward: 5′TCTAGAGTAGATTCAGAAAGCAGCCACCT3′  Reverse: 5′GGATCCGCTGCTGTTGTAGTGGTAGAAGTTT3′ | Subcellular localization |
| *PnWRKY15* | Forward: 5′GAATTCTGACTGATACTTCTCCCAAGTCT3′  Reverse: 5′GGATCCTCATGCATGCCTCCTAAGGG3′ | Overexpression |
| *PnWRKY15* | Forward: 5′GGGGACAAGTTTGATCAAAAAAGCAGGCTGCATGACTGATACTTCTCCCAAGTCT3′  Reverse: 5′GGGGACCACTTTGTACAAGAAAGCTGGGTCTCATGCATGCCTCCTAAGGGAAT3′ | RNAi |
| PPnOLP1 | GSP1: 5′GGTGAGGGTATGGAGGGCGAAGC3′  GSP2: 5′GGTGAAGGGGCAGTTGTTAACTACGGT3′ | Genome-walking |
| *PnWRKY15* | Forward: 5′GTCGACATGACTGATACTTCTCCCAAGTCT3′  Reverse: 5′GGATCCTGCATGCCTCCTAAGGGAAT3′ | EMSA |
| Probe | TACCGTGAAGAAATATATTTTTCTTTTGACCGACTATGGTGAGGGATGAA | EMSA |
| Mutant probe | TACCGTGAAGAAATATATTTTTCTTTTACCCGACTATGGTGAGGGATGAA | EMSA |
| PPnOLP1 | Forward: 5′AAGCTTACATCCTACCGTGAAGA3′  Reverse: 5′GGATCCGAGGAGTGAAAGGGCAT3′ | Y1H |
| *PnWRKY15* | Forward: 5′GAATTCATGACTGATACTTCTCCCAAGTCT3′  Reverse: 5′GGATCCTCATGCATGCCTCCTAAGGG3′ | Y1H |
| PPnOLP1 | Forward: 5′AGTACTACATCCTACCGTGAAGA3′  Reverse: 5′TCTAGAGAGGAGTGAAAGGGCAT3′ | Co-expression |

Table S5 The primers for qRT-PCR

| Target gene | Primer sequences |
| --- | --- |
| *PnWRKY15* | Forward: 5′CCTAACACTGAAGAATCCACCTC3′  Reverse: 5′GCTCTGAGACTGACCACTCCACTT3′ |
| *PnAOS* | Forward: 5′AATGGCGGCGATGGAA3′  Reverse: 5′ACGGTTGGAACCCGAATAA3′ |
| *PnPR-1* | Forward: 5′ACCTTGCCTATGGCTTCCCT3’  Reverse: 5′TGTTACACCTCGCCCTACCG3’ |
| *PnMYC2* | Forward: 5′CAAGAGACCTCGGAAGCGG3′  Reverse: 5′GCAGTTCGGTTATGTAAGCAAT3′ |
| *PnCHI* | Forward: 5′CCACAGGGTAACAAGCCATCG3’  Reverse: 5′AAGAATCCAATCCTATCCTCCACTC |
| *PnOLP* | Forward: 5′GCCACTTGTCCCGTTAGCTTG3′  Reverse: 5′AATCCGAATAGTTGGAAGCCTTG3′ |
| *PnACT2* | Forward: 5′TCCAAGGGTGAATATGATGAATCG3′  Reverse: 5′AACCTCTCCAAAGAGAATTTCTGAGT3′ |
| *NtPR1* | Forward: 5′AGAACCTTTGACCTGGGACGAC3′  Reverse: 5′ATCCAACACGAACCGAGTTACG3′ |
| *NtCHI* | Forward: 5′ACGGACCTTGTGGAAGAGCCAT3′  Reverse: 5′ACCAAATCCAGGGAGACGATTG3′ |
| *Ntosmotin* | Forward: 5′CGACTATCGAGGTCCGAAACAAC3′  Reverse: 5′ACGTACCCCTACCAGCAGCATT3′ |
| *NtMYC* | Forward: 5′GGAACAATCGAGTTTACAGAGATCAA3′  Reverse: 5′CAGCAAGTTGATTAAGAAACCTAGC3′ |
| *NtAOC* | Forward: 5′AAGAAGAGAATTGGAATAACGGCT 3′  Reverse: 5′GATCCCTGAACGGCGATGT 3′ |
| *NtPACX* | Forward: 5′AAGCAGCTAAGTTAAGGCATTTTGTA3′  Reverse: 5′GTTCAGTTTGAGCGTAGCACCCA3′ |
| *NtAOS* | Forward: 5′CCACCAGTTGCTTCTCAATACGG3′  Reverse: 5′GAACTCATCGGGTCGGTCAAA3′ |
| *NtJMT* | Forward: 5′TTGGGTACTGAAGCAAGGACAGC3′  Reverse: 5′GCTCCTCCCCATTAACGACAAC3′ |
| *NtOPR* | Forward: 5′CGTTCATTTGTAGTGGCGGAT3′  Reverse: 5′CCTCATAACCAAATCAGGATTAGAAA3′ |
| *NtACT* | Forward: 5′TCCCATTGAGCATGGAATAGTAAGC3′  Reverse: 5′TACATGGCAGGTACATTGAAAGTCT3′ |
