# Supplementary material for: Panax notoginseng transcription factor WRKY15 modulates resistance to Fusarium solani by up-regulating osmotin-like protein expression and inducing JA/SA signaling pathways
Source: BMC Plant Biol. 2023 Jul 17;23:362. doi: 10.1186/s12870-023-04373-x (PMC10351173; doi:10.1186/s12870-023-04373-x)
Supplement: Supplementary file 2 — Supplementary Material 2 [file 12870_2023_4373_MOESM2_ESM.docx]

The sequence of PPnOLP1

>PPnOLP1

CCTCATTATTCTAAGACAAATTAGAAATTCTAACATCCTACCGTGAAGAAATATATTTTTCTTTTGACCGACTATGGTGAGGGATGAAAAAGCAAATTAAAACTTTAAAAAATAAAATTAATAGTTGTTTTTTTTTTTTGGTAAAATAAAAGATCGTCTTAAGGCGGAAGGTGCATTGTTTATTGCTTAATGGTTCATATTTGAGATTAAAATGGTCCATAACTTTTTTGTATTCAATTTGTGACCAGCATTTTGTGATATGTGGATTCATTGGACACATGGGAAGCTGGGAGTTGAGTCCAAATTGTTTGGATATCCATAACAGAAGAAAACCGACACAGGCAGGCTCCCTTATCCATATTGGCGCTACATTTGCACGATAAAAAATTACATTTTGAATAAAGTAGGAGTATTATACAAATAATTCTAACTTTTTAATATATATATATATATCATTTCAATAAAATTGTTCGAATTTGACAATGACACAAGGGCCCAAGATAAAAACAGAAAACAGCTAAAAGCAAACTAAAGGCTACGTGAATTTCGATCAGATTTTAAAAGAATGACATGCTTTGGATATTCGGCTTTATGCTTTTAGGTTACATTTTTCCTTTGCTAGTAGTAGTATTTATCTTTTACTTTACTTTTAACATATACTAGTATAATATAGCTTACAATAACTTATAACTTGGCATGCATTAGGTTAGGTTCTAGCTGGCTAGAATTCGGGTAAGATTAACGATATAAACGTAAAAATATTATTTTCACGTAATCCTTTTTTACATAAAGAAAATTCACATTCTATTACAACAGGCTAATAAACAAAAGAATTATTTTTTGAAAAATATTCTATTATATTTTTTCTGTTTTTACATAATACCAAACTTTAAACAATCTCAAATGAACAAAATATTTCCTCAACAAATAAAAAAGGACACTAAATATCAGTTTTTCACAAAAAATAGCCTAAGATCAACATGCCCTTTCACTCCTCTATATATAACAACTCAATCCCCCGTCCCATTCTT
