# Supplementary material for: Panax notoginseng transcription factor WRKY15 modulates resistance to Fusarium solani by up-regulating osmotin-like protein expression and inducing JA/SA signaling pathways
Source: BMC Plant Biol. 2023 Jul 17;23:362. doi: 10.1186/s12870-023-04373-x (PMC10351173; doi:10.1186/s12870-023-04373-x)
Supplement: Supplementary file 3 — Supplementary Material 3 [file 12870_2023_4373_MOESM3_ESM.docx]

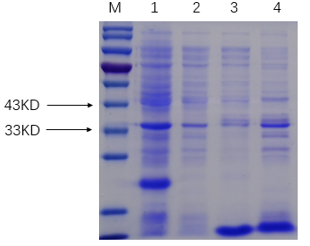


Fig. S1 Solubility analysis of PnWRKY15 recombinant protein

M: protein maker; 1: Empty pET-32a vector was transformed into Rosetta plus IPTG induced protein; 2: pET32a-*PnWRKY15* vector was transformed into *E. coli* Rosseta protein without IPTG induction; 3: Supernatant after PnWRKY15 inclusion body protein crushing; 4: PnWRKY15 inclusion body protein is precipitated after crushing.


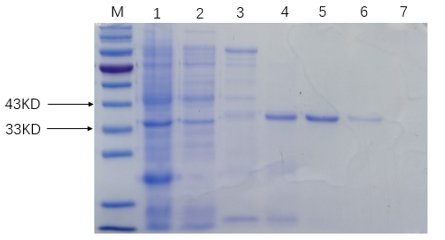


Fig. S2 Purification of PnWRKY15 recombinant protein

M: protein maker; 1: Empty pET-32a vector was transformed into Rosetta plus IPTG induced protein; 2: pET32a-*PnWRKY15* vector was transformed into escherichia coli Rosetta protein without IPTG induction; 3, 4, 5, 6 and 7 were eluents Ⅰ, Ⅱ, Ⅲ, Ⅳ, Ⅴ, respectively.
